# Enteric infections and management practices among communities in a rural setting of northwest Ethiopia

**DOI:** 10.1038/s41598-023-29556-2

**Published:** 2023-02-09

**Authors:** Zemichael Gizaw, Negesu Gizaw Demissie, Mulat Gebrehiwot, Bikes Destaw, Adane Nigusie

**Affiliations:** 1 Department of Environmental and Occupational Health and Safety, Institute of Public Health, College of Medicine and Health Sciences, University of Gondar, Gondar, Ethiopia; 2 Department of Medical Nursing, School of Nursing, College of Medicine and Health Sciences, University of Gondar, Gondar, Ethiopia; 3 Department of Health Education and Behavioral Sciences, Institute of Public Health, College of Medicine and Health Sciences, University of Gondar, Gondar, Ethiopia

**Keywords:** Microbiology, Diseases, Gastroenterology, Risk factors

## Abstract

Infections with enteric pathogens have a high mortality and morbidity burden, as well as significant social and economic costs. Poor water, sanitation, and hygiene (WASH) conditions are the leading risk factors for enteric infections, and prevention in low-income countries is still primarily focused on initiatives to improve access to improved WASH facilities. Rural communities in developing countries, on the other hand, have limited access to improved WASH services, which may result in a high burden of enteric infections. Limited information also exists about the prevalence of enteric infections and management practices among rural communities. Accordingly, this study was conducted to assess enteric infections and management practices among communities in a rural setting of northwest Ethiopia. A community-based cross-sectional study was conducted among 1190 randomly selected households in a rural setting of northwest Ethiopia. Data were collected using structured and pretested interviewers-administered questionnaire and spot-check observations. We used self-reports and medication history audit to assess the occurrence of enteric infections among one or more of the family members in the rural households. Multivariable binary logistic regression model was used to identify factors associated with enteric infections. Statistically significant association was declared on the basis of adjusted odds ratio with 95% confidence interval and *p* value < 0.05. Out of a total of 1190 households, 17.4% (95% CI: 15.1, 19.7%) of the households reported that one or more of the family members acquired one or more enteric infections in 12 months period prior to the survey and 470 of 6089 (7.7%) surveyed individuals had one or more enteric infections. The common enteric infections reported at household-level were diarrhea (8.2%), amoebiasis (4.1%), and ascariasis (3.9%). Visiting healthcare facilities (71.7%), taking medications without prescriptions (21.1%), and herbal medicine (4.5%) are the common disease management practices among rural households in the studied region. The occurrence of one or more enteric infections among one or more of the family members in rural households in 12 months period prior to the survey was statistically associated with presence of livestock (AOR: 2.24, 95% CI:1.06, 4.75) and households headed by uneducated mothers (AOR: 1.62, 95% CI: (1.18, 2.23). About one-fifth of the rural households in the studied region reported that one or more of the family members had one or more enteric infections. Households in the study area might acquire enteric infections from different risk factors, mainly poor WASH conditions and insufficient separation of animals including their feces from human domestic environments. It is therefore important to implement community-level interventions such as utilization of improved latrine, protecting water sources from contamination, source-based water treatment, containment of domestic animals including their waste, community-driven sanitation, and community health champion.

## Introduction

**Figure 1:**
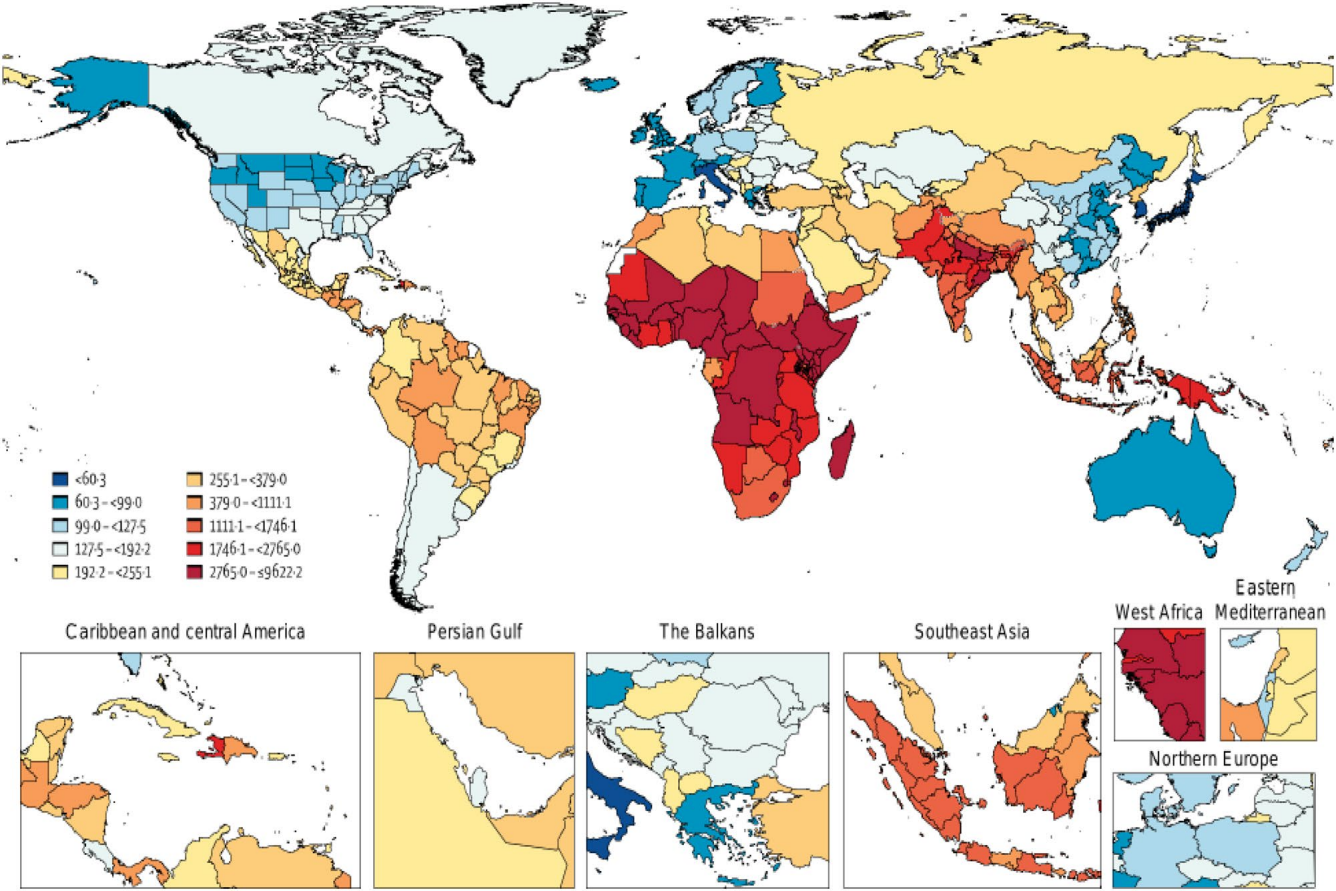
Age-standardized DALY rates (per 100 000) by location, both sexes combined, 2019 (source: https://www.healthdata.org/results/gbd_summaries/2019/enteric-infections-level-2-cause)^2^.

Enteric diseases are caused by micro-organisms such as viruses, bacteria and parasites that cause intestinal illness. The illnesses most frequently result from consuming contaminated food or water, and some can spread from person to person ^1^. Infections with enteric pathogens have a high mortality and morbidity burden, as well as significant social and economic costs. In 2019, there were 6.60 billion incident cases and 98.8 million prevalent cases of enteric infections, resulting in 1.75 million deaths, and 96.8 million disability-adjusted life years (DALYs), where the problem is disproportionately high in the global south (low to middle-income countries)^2^. Figure 1 illustrates age-standardized DALY rates (per 100 000) by location.

Environmental factors such as poor water, sanitation, and hygiene (WASH) conditions are among the leading risk factors for enteric infections. Poor WASH conditions are likely to lead to contamination of hands, water, foods, and the environment, resulting in the spread of fecal organisms^3,4^. When pathogens are transmitted via environmental media, a dynamic interaction between pathogen, host, and environment determines whether the host becomes infected or develops symptoms. Pathogen phenotypes, host physiology, and environmental factors can all lead to an increase or decrease in infection risk. Furthermore, people differ in terms of physiologic or immune factors and likelihood of exposure that alter the likelihood of infection^5–8^.

Obviously, different strategies are recommended to prevent and reduce enteric infections and their consequences in various parts of the world. Prevention in low-income countries remains primarily focused on initiatives to ensure (i) access to safe drinking water and sanitation, (ii) food safety, and (iii) environmental protection^9^. Sanitation aims to prevent contamination of the environment by excreta and, therefore, to prevent transmission of pathogens that originate in feces of an infected person^10,11^. There are numerous technologies and methods available to accomplish this, including sophisticated and high-cost methods such as waterborne sewage systems and simple low-cost methods such as the cat method, which involves digging a hole and covering feces with soil after defecation^11,12^. Water supply aims to provide the community with safe or clean and adequate water from accessible and affordable improved water sources^13^. Furthermore, because universal safe and reliable water supply remains an elusive goal for the vast majority of the world’s population, household-level water treatment (HWT) has been proposed as a stopgap solution to provide safer drinking water at the point of use^14,15^. Food hygiene interventions, such as the promotion of reheating foods, preventing contact with flies and hand washing before feeding intends to keep food from becoming contaminated^16,17^. To this end, awareness programs will continue to play an important role in preventing the spread of infections. Furthermore, empowering society to play a more active role in the prevention of infections such as water and food-borne illnesses is critical^18,19^. However, rural communities in developing countries have limited access to improved WASH services which may lead to high burden of enteric infections^20^. Limited information also exists about the prevalence of enteric infections and management practices among rural communities. Accordingly, this study was conducted to assess enteric infections and management practices among communities in a rural setting of northwest Ethiopia.

## Methods

### Study design and setting

A community-based cross-sectional study with structured observation was conducted among rural households in Central and North Gondar administrative zones of the Amhara national regional state, Ethiopia in May 2016. Central Gondar zone covers thirteen districts and North Gondar zone covers seven districts. The total population residing in Central Gondar is estimated to be 2,896,928 and it is estimated to be 912,112 in North Gondar zone^21^.

### Sample size calculation and sampling procedures

The sample size (i.e., 1210 rural households) was calculated using single population proportion formula and the target households were included in the study using systematic random sampling technique. The sample size calculation and sampling procedures are described in more detail elsewhere^22^.

### Data collection tools and procedures

Data were collected using a structured and pretested interviewers-administered questionnaire and spot-check observations (supplementary file 1). The questionnaire and observation checklists were prepared based on a review of relevant literature. The questionnaire was first prepared in English language and translated to the local Amharic language by two native Amharic speakers fluent in English, and back-translated into English by two independent English language experts fluent in Amharic to check consistency. The questionnaire was organized in to three parts: (i) socio-demographic information; (ii) WASH conditions; and (iii) enteric infections, management practices, and associated deaths. Data were collected by public health experts and the data collection was closely supervised by field supervisors.

### Measurement of variables

The primary outcome variable for this study was prevalence of enteric infections among one or more of the family members in rural households of northwest Ethiopia. Prevalence of enteric infections was defined as the occurrence of one or more gastrointestinal health problems (such as cholera, typhoid fever, salmonellosis, diarrhea, amoebiasis, giardiasis, ascariasis, hookworm, and schistosomiasis) in 12 months period prior to the survey. The household head was asked about the occurrence of the aforementioned health problems among any of the family members. To make it clear to the participants, we used the local names for each disease. Furthermore, we checked the medication history of individuals who visited health facilities to identify the specific diseases confirmed by the physicians.

Latrine facilities (one of the predictors) were taken as “improved” if the household used clean, well-maintained, and unshared latrine facilities such as pit latrines (including ventilated pit latrines) which are accessible at night use and has functional handwashing facilities^23^. Drinking water sources (the other predictor) were taken as “improved” if the sources have the potential to deliver safe water by nature of their design and construction, and include pipeline, public taps, protected wells, protected springs, and protected rain catchments^24^.

### Data processing and analysis

Data were entered using EPI-INFO version 3.5.3 statistical package and exported into Statistical Package for Social Sciences (SPSS) version 20 for further analysis. Descriptive statistics, such as frequency and percentage were used to present the data. We included predictors to the multivariable binary logistic regression model from the literature regardless of their bivariate *p *value to identify factors associated with enteric infections among rural households. Statistically significant association was declared on the basis of adjusted odds ratio (AOR) with 95% confidence interval (CI) and *p *values < 0.05. Model fitness was check using Hosmer and Lemeshow goodness-of-fit test.

### Ethics approval and consent to participate

Ethical clearance was obtained from the Institutional Review Board of the University of Gondar (reference number: V/P/RCS/05/1520/2016). There were no risks due to participation and the collected data were used only for this research purpose with complete confidentiality. Written informed consent was obtained from household heads. All the methods were carried out in accordance with relevant guidelines and regulations.

## Results

### Sociodemographic characteristics

Of a total of 1210 rural households, 1190 households participated in the current study, with a response rate of 98.3%. A total of 6089 individuals (3187 males and 2902 females) were surveyed in 1190 households and 513 (43.1%) of the households had more than five family members. Over three quarter, 888 (75.3%) of the female heads did not receive formal education and 643 (59.3%) of the male heads did not attend formal education. The vast majority, 1123 (95.2%) of the female heads were farmers by their occupation and 1045 (96.3%) of the male heads were also farmers. Majority of the households, 1113 (85.1%) had one or more children, out of which 467 (39.2%) had children under the age of six years. One hundred and forty-seven (12.4%) of the households reported that one or more of the family members had vision impairment and 52 (4.4%) of the households reported that one or more of their family members had difficulty of mobility. One thousand and eighty-one (90.8%) of the households had one or more domestic animals in which their excrement is not contained from the living environment (Table 1).

**Table 1:** Sociodemographic characteristics of households (n = 1190) in a rural setting of northwest Ethiopia, May 2016.

Sociodemographic characteristics	Frequency	Percent
Family size of households (n = 1190)		
< 5	677	56.9
> 5	513	43.1
Number of individuals surveyed in 1190 households (n = 6089)		
Male	3187	52.3
Female	2902	47.7
Maternal education (n = 1180)		
No formal education	888	75.3
Attend formal education	292	24.7
Paternal education (n = 1085)		
No formal education	643	59.3
Attend formal education	442	40.7
Maternal occupation (n = 1180)		
Farmer	1123	95.2
Merchant	35	3.0
Government employee	8	0.7
Student	3	0.3
Daily Laborer	11	0.9
Paternal occupation		
Farmer	1045	96.3
Merchant	22	2.0
Government employee	9	0.8
Daily Laborer	9	0.8
The household has a child/child (n = 1190)		
< 1 year old children	75	6.3
1–5 years old children	392	32.9
6–10 years old children	228	19.2
11–14 years old children	318	26.7
The household has no children	177	14.9
One or more of the family members have vision impairment		
Yes	147	12.4
No	1043	87.6
One or more of the family members have difficulty of mobility		
Yes	52	4.4
No	1138	95.6
Th household has livestock*		
No	109	9.2
Ox	839	70.5
Cow	858	72.1
Sheep	567	47.6
Goat	128	10.8
Horse	355	29.8
Mule	90	7.6
Donkey	326	27.4
Hen	47	3.9

*Households had two or more livestock.

### WASH conditions

Five hundred and sixty-five (47.5%) of the households reported that they received WASH education and 967 (81.3%) of the households reported that they have been regularly supervised by health professionals. The vast majority, 1100 (92.4%) of the households had no access to improved sanitation facilities. About one-fifth, 233 (19.6%) of the households collected drinking water from unimproved sources. Moreover, food items in 312 (26.2%) of the households were not protected from pets and mechanical vectors such as common house flies had been observed in food storage areas among 238 (20.0%) of the households (Table 2).

**Table 2:** Water, sanitation and hygiene conditions of households (n = 1190) in a rural setting of northwest Ethiopia, May 2016.

WASH conditions	Frequency	Percent
WASH education		
Yes	565	47.5
No	625	52.5
Health professionals’ regular supervision		
Yes	967	81.3
No	223	18.7
Access to improved latrine		
No	1100	92.4
Yes	90	7.6
Drinking water sources		
Unimproved	233	19.6
Improved	957	80.4
Food items are protected from pets		
Yes	878	73.8
No	312	26.2
Presence of mechanical vectors in food storage areas		
Yes	238	20.0
No	952	80.0

### Enteric infections and management practices

**Figure 2:**
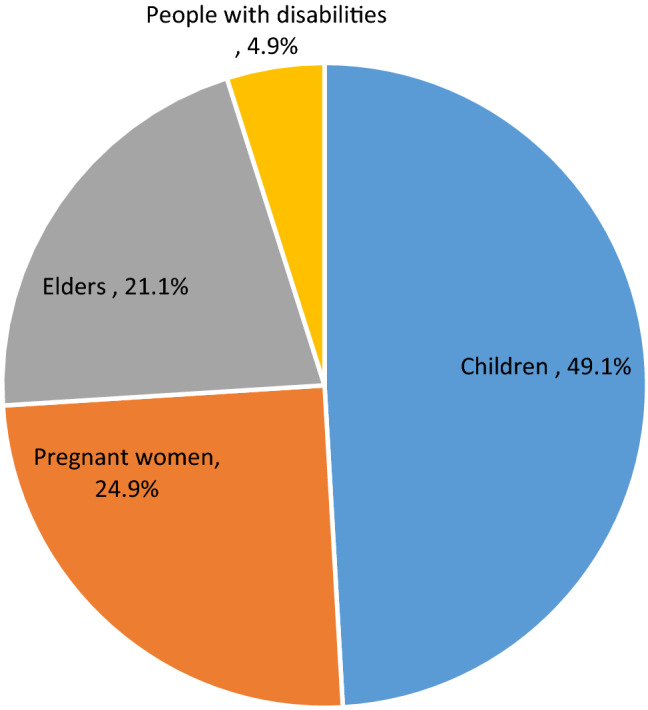
Distribution of enteric infections among vulnerable groups of the community in a rural northwest Ethiopia, May 2016 (note: elders were family members aged above 64 years).

Out of a total of 1190 households, 207 (17.4%) (95% CI: 15.1, 19.7%) of the households reported that one or more of the family members acquired one or more enteric infections in 12 months period prior to the survey and 470 of 6089 (7.7%) individuals had one or more enteric infections, of which 231 of 470 (49.1%) were children under the age of 14 years (Fig. 2). The common enteric infections reported at household-level were diarrhea [97 (8.2%)], amoebiasis [49 (4.1%)], and ascariasis [46 (3.9%)]. In the current study 337 of 470 (71.7%) and 98 of 470 (21.1%) individuals who had one or more enteric infections visited healthcare facilities and took medications without prescriptions, respectively to manage the infection/s. Out of 470 individuals who acquired one or more enteric infections, 7 (1.5%) of them died due to the infection/s (Table 3).

**Table 3:** Enteric infections, management practice, and associated deaths among communities in a rural northwest Ethiopia, May 2016.

Enteric infections reported	Frequency	Percent
Common enteric infections reported at household level (n = 1190) *		
Diarrhea†	97	8.2
Amoebiasis‡	49	4.1
Ascariasis‡	46	3.9
Tapeworm‡	26	2.2
Cholera‡	22	1.8
Typhoid fever‡	18	1.5
Salmonellosis‡	17	1.4
Giardiasis‡	10	0.8
Hookworm‡	3	0.3
Schistosomiasis‡	2	0.2
Management of enteric infections (n = 470)		
Visit healthcare facilities	337	71.7
Taking medication without prescription	98	21.1
Use traditional medicine	21	4.5
No action	16	3.4
Number of deaths reported due to above disease/s (n = 470)		
Male	5	1.1
Female	2	0.4
No death	463	98.5

*There were households who reported two or more enteric infections.

^†^Self-reported.

^‡^Checked from medication history.

### Factors associated with enteric infections

Access to improved latrine facilities, drinking water sources, mechanical vectors seen in food storage area, protection of food items from pets, presence of livestock, health education, regular health supervision, maternal education, and paternal education were the variables entered in to the adjusted model. The occurrence of one or more enteric infections among one or more of the family members in rural households in 12 months period prior to the survey was statistically associated with presence of livestock and maternal education. The odds of acquiring one or more enteric infections among one or more of the family members in rural households was 2.24 times higher among households who owned one or more livestock compared with households with no live stock (AOR: 2.24, 95% CI:1.06, 4.75). Similarly the odds of enteric infections among rural households was 1.62 times higher among households headed by mothers who did not attend formal education compared with their counterparts (AOR: 1.62, 95% CI: (1.18, 2.23) (Table 4).

**Table 4:** Factors associated with enteric infections among households (n = 1190) in a rural setting of northwest Ethiopia, May 2016.

Variables	Enteric infections		COR with 95% CI	AOR with 95% CI
	Yes	No		
Access to improved latrine facilities				
No	193	907	1.16 (0.64, 2.09)	1.30 (0.70, 2.44)
Yes	14	76	1.0	1.0
Drinking water sources				
Unimproved	38	195	0.91 (0.62, 1.34)	0.89 (0.60,1.33)
Improved	169	788	1.0	1.0
Vectors seen in food storage area				
Yes	43	195	1.06 (0.73, 1.54)	1.09 (0.74, 1.60)
No	164	788	1.0	1.0
Food items protected from pets				
Yes	155	723	1.0	1.0
No	52	260	0.93 (0.66, 1.32)	0.87 (0.60, 1.25)
Presence of livestock				
No	9	100	1.0	1.0
Yes	198	883	2.49 (1.24, 5.01)	2.24 (1.06, 4.75)*
Health education				
Yes	99	466	1.0	1.0
No	108	517	0.98 (0.73, 1.33)	1.10 (0.79, 1.55)
Regular supervision				
Yes	173	794	1.0	1.0
No	34	189	0.83 (0.55, 1.23)	0.85 (0.55, 1.33)
Maternal education				
No formal education	161	727	1.18 (0.83, 1.69)	1.62 (1.18, 2.23)**
Attend formal education	46	246	1.0	1.0
Paternal education				
No formal education	112	531	0.89 (0.65, 1.21)	0.79 (0.56, 1.10)
Attend formal education	85	357	1.0	1.0

*statistically significant at *p* < 0.05,** statistically significant at *p* < 0.01, Hosmer and Lemeshow test: 0.238, *AOR* Adjusted odds ratio, *CI* Confidence interval, and *COR* Crude odds ratio.

## Discussion

This is a community-based cross-sectional study conducted to assess enteric infections among households in a rural setting of northwest Ethiopia and found that 17.4% (95% CI: 15.1, 19.7%) of the households reported that one or more of the family members acquired one or more enteric infections in 12 months period prior to the survey. Out of 6089 individuals surveyed in all the households, 7.7% individuals had one or more enteric infections. The prevalence of enteric infections reported in the current study is lower than findings of studies in rural Lao, 98.3%^3^ and in a rural area and urban slum of Vellore, India, 31%^25^. This low prevalence is due to limitations of the methods we used, i.e., self-report and medication history audit. The self-reported data may not be reliable since the study subjects may make the more socially acceptable answer rather than being truthful and they may not be able to assess themselves accurately. Moreover, we checked the medication history to identify the specific diseases and we asked the household heads to tell us the name of the diseases confirmed by the physicians for individuals who visited health facilities. These methods may underestimate the prevalence of enteric infections since the health seeking behavior of the rural communities in the study areas is very low.

According to this study, the most common disease management practices among rural households in the studied region are visiting healthcare facilities, taking medications without a prescription, and using herbal medicine. Since Ethiopia has notable progress in expanding healthcare facilities at all levels, access to healthcare services for the rural communities is rapidly increasing over the past decades. This results in an increase in the use of healthcare services^26,27^. Moreover, there is credible community health promotion and public involvement through the health extension program^28,29^. All these help the rural communities to use modern medicine for disease management in the area. On the other hand, there is still healthcare disparities in the rural communities, which lead the communities to use herbal medicines^30,31^. Moreover, since there is no strong regulatory system in Ethiopia, over the counter or non-prescription sale of antibiotics is a common practice in the country^32,33^. Unless this practice is resolved, it may create critical public health problems, such as drug resistance^34,35^.

The occurrence of one or more enteric infections among one or more of the family members in rural households in 12 months period prior to the survey was statistically associated with presence of livestock. This finding is in agreement with findings of other studies^36–40^. The association between enteric infections and domestic animals can be due to the fact that animal husbandry and keeping practices in the rural communities could result human contact with animals or animal feces and contamination of the living environment. Pathogens from animal excreta could reach to humans via fecally contaminated water, soil, foods, and hands^41–43^. Animal excreta from contaminated living environment could reach to water sources via the help of flooding and could result fecal contamination of water sources which leads exposure to enteric pathogens through drinking of contaminated water or ingestion of foods prepared by fecally contaminated water^41,44^. Hands could also be contaminated while the rural communities performed their day-to-day tasks in the fecally contaminated living environment which help pathogens to reach to humans via a direct hand-to-mouth contact or via food contamination from unwashed hands^41,45,46^. Presence of animal excreta in the living environment on the other hand creates favorable conditions for mechanical vectors which may carry diseases causing pathogens from animal excreta to food items^41,47,48^. Moreover, food items could be contaminated with pathogens when animals get entered in to the living quarter and accessed prepared and stored foods via their saliva, hairs or fissures, and feet^49^.

This study also found that enteric infections among one or more of the family members in rural households in 12 months period prior to the survey was statistically associated with maternal education which is in line with findings of other studies^50,51^. The association between education and enteric infection can be justified that educated mothers may have increased awareness about the transmission and prevention methods of infections, whereas uneducated mothers may not have awareness about diseases transmission and prevention methods. Education is likely to enhance household health and sanitation practices and encourages healthy behavior changes^52–54^.

As limitations, we couldn’t identify predictors of enteric infections at the individual-level because we have no individual-level data for most of the variables. The self-reported data may not be reliable since the study subjects may make the more socially acceptable answers rather than being truthful and they may not be able to assess themselves accurately, which might result reporting bias. Moreover, a larger study is required to ensure generalizability.

## Conclusion

About one-fifth of the rural households in the studied region reported that one or more of the family members had one or more enteric infections in 12 months period prior to the survey and about half of the infected individuals were children under the age of 14 years. Households in the study area might acquire enteric infections from different risk factors, mainly poor WASH conditions and insufficient separation of animals including their feces from human domestic environments. Moreover, more than one-fifth of the individuals who acquired one or more enteric infections took medications without prescription, which may create critical public health problems, such as drug resistance unless regulated. Community-driven sanitation interventions such as promotion of improved latrine construction and utilization, containment of domestic animals including their waste, and controlled disposal of domestic wastewater and rubbish should be critically implemented in the area. Preventing contamination of drinking water at its source (such as water source protection and source-based water treatment) and at point of use (such as safe water storage and home-based water treatment) is very important. Furthermore, community health champion, health and sanitation education, and proper use of medications should be in placed to improve health, sanitation, and health seeking behaviors of the community.

## Supplementary Information

Supplementary Information.

## Data Availability

Data will be made available upon requesting the primary author.
