# Supplementary material for: Enteric infections and management practices among communities in a rural setting of northwest Ethiopia
Source: Sci Rep. 2023 Feb 9;13:2294. doi: 10.1038/s41598-023-29556-2 (PMC9911403; doi:10.1038/s41598-023-29556-2)
Supplement: Supplementary file 1 — Supplementary Information. [file 41598_2023_29556_MOESM1_ESM.doc]

| **Questionnaire Identification Code:** | **District:** | **Kebele:** | **Household:** |
| --- | --- | --- | --- |

**Consent Form**

Hello, I am ________________________ working with a research team from University of Gondar. We are contacting you to collect information for a study conducted to “*assess enteric infections and management practices among communities in a rural setting of northwest Ethiopia*”. This questionnaire is prepared to collect sociodemographic information, health and sanitation messages, WASH conditions, common enteric infections and management practices among rural households.

Thank you so much for agreeing to be interviewed for this project. All the information we got from you will be completely confidential and coded with unique number. Your name will not be written down and will never be used in connection with any of the information you provide. If you do not want to answer all or some of the questions, you have the right to refuse participation at any time. However, we would greatly appreciate your help in responding to this questionnaire. This questionnaire is expected to be completed in 30 - 40 minutes.

Do I have your permission to continue?

1. **Yes,** continue your interview with thanks
2. **If no,** skip to the next participant by writing reasons for his/ her refusal;

____________________________________________________________________________

**Data Collector:** Name: ____________________Signature:___________Date: __________

Time started _______Time completed_______

**Result of interview**:

1. Completed 3. Refused

2. Respondent not available 4. Incomplete

**Approval from Supervisor:**

Name: ___________________ Signature: ___________ Date: ________________

| **Part 1: Socio-demographic information** | | | | | | | | | | | |
| --- | --- | --- | --- | --- | --- | --- | --- | --- | --- | --- | --- |
| 1 | List of Family member | Sex | Age | | | Education | Marital status | Occupation | | Family Head | |
|  |  |  | | |  |  |  | |  | |
|  |  |  | | |  |  |  | |  | |
|  |  |  | | |  |  |  | |  | |
|  |  |  | | |  |  |  | |  | |
|  |  |  | | |  |  |  | |  | |
|  |  |  | | |  |  |  | |  | |
|  |  |  | | |  |  |  | |  | |
| **List of Family member**   1. Father 2. Mother 3. Son (if more than one wrte S1, S2, S3, …) 4. Daughter (if more than one write D1, D2, D3 …) 5. Relative (if more than one, write R1, R2, …) 6. House maid 7. Grandfather 8. Grand mother | | Sex   1. Male 2. Female | | Head of the family   1. Father 2. Mother 3. Grand father 4. Grand mother 5. Old son 6. Old daughter | | **Marital status**   1. - Married 2. Single 3. Divorced 4. Separated 5. Widowed 6. Cohabited 7. Not applicable | | **Educational status**   1. Pre-school 2. Not read and write 3. Read and write 4. Primary school 5. Secondary school 6. 12 complete 7. College/University | | **Occupation**   1. Farmer 2. Merchant 3. Civil servant 4. Student 5. NA | |
| 2. | Presence of livestock | | | | | 1. Yes 2. No | | | | Skip | |
|  | Is /are there person/s who has/ have vision problem? | | | | | 1. Yes (if ‘Yes’ degree of problem _____________________________) 2. No | | | |  | |
| 4. | Is /are there person/s who has/ have difficulty of mobility or squatting? | | | | | 1. Yes 2. No | | | |  | |
| **Part 2: Health information and supervision** | | | | | | | | | | | |
|  | Have you discused about health, hygiene, sanitation and other health issues with the family members? | | | | | 1. Yes 2. No | | | |  | |
|  | Does the health extension worker or other health professional closely supervise you? | | | | | 1. Yes 2. No | | | |  | |
|  | Have you received health or WASH education in the last 3 months? | | | | | 1. Yes 2. No | | | |  | |
| **Part 3: Human excreta management** | | | | | | | | | | | |
|  | Defecation practice of household members? (Observe if it is latrine) | | | | | 1. In the open field 2. Latrine | | | |  | |
|  | If it is latrine for # 1, what kind of toilet facility doesthis household use? | | | | | 1. Pit latrine 2. VIP latrine 3. Hanging latrine | | | |  | |
|  | If it is latrine for # 1, is the toilet facility used by day and night? | | | | | 1. Day and night 2. Daytime only 3. Nighttime only | | | |  | |
|  | If it is latrine for # 1, is the facility cleaned? | | | | | 1. Yes 2. No | | | |  | |
|  | If it is latrine for # 1, is the area around the latrine is free of excreta? **(Observe it**) | | | | | 1. Yes 2. No | | | |  | |
|  | If it is latrine for # 1, does the track to the latrine indicate it is not used or covered with grass? **(Observe it**) | | | | | 1. Yes 2. No | | | |  | |
|  | If it is latrine for # 1, what is the condition of latrine?  **(Observe it)** | | | | | 1. Well maintained 2. The superstructure needs maintainance 3. The slab needs maintainance | | | |  | |
|  | Is there functional hand washing facility (filled with water and soap is available) around the latrine? **(Observe it**) | | | | | 1. Yes 2. No | | | |  | |
| **Part 4: Water supply and food safety** | | | | | | | | | | | |
|  | What is the main source of water for your household? | | | | | 1. Piped line 2. Public taps/tapstand/standpipes 3. Protected hand pump/borehole/tubewell 4. Unprotected hand pump/borehole/tubewell 5. Protected dug well 6. Unprotected/open dug well 7. Protected spring 8. Unprotected spring 9. Protected Rain catchment 10. Unprotected Rain Catchment 11. Surface water (river, dam, lake, ponds, creeks, canal, etc.)   13 Others, specify ____________________ | | | |  | |
|  | Does your water source provide water throughout the year? | | | | | 1. Yes 2. No | | | |  | |
|  | Time taken to fetch water (round trip including queing time) in minute? | | | | | _______________________________ | | | |  | |
|  | How many liters of water do the entire households consume/use per day? (note that standard 1 Jerry Can = 20L) | | | | | _________________________ | | | |  | |
|  | Which specific water container do you usually store your drinking water? (more than one answer is posible) | | | | | 1. Jerry Can 2. Bucket 3. Clay pots 4. Drum/barrel 5. Others, specify ______________ | | | |  | |
|  | Observe the condition of the water containers for collection and storage. | | | | | 1. Clean 2. Not clean 3. Properly covered 4. Not covered | | | |  | |
|  | What do you use to wash food utensils? | | | | | 1. Water only 2. Soap and water 3. Ash and water | | | |  | |
|  | Where you store foods and food utensils? | | | | | 1. On the floor 2. On shelf made from wood and mud | | | |  | |
|  | Vectors or rodents are seen in food storage area | | | | | 1. Yes 2. No | | | |  | |
|  | Food and food utensils are protected from pets | | | | | 1. Yes 2. No | | | |  | |
| **Part 5: Enteric infections and management** | | | | | | | | | | | |
|  | In the last 12 months, has anyone in your family had the following diseases? | | | | What do you think about the cause? | | | | Methods of confirmation (self-report or medication history) | | |
|  | Diarrhea | | | |  | | | |  | | |
|  | Salmonella | | | |  | | | |  | | |
|  | Cholera | | | |  | | | |  | | |
|  | Ascariasis | | | |  | | | |  | | |
|  | Amoebiasis | | | |  | | | |  | | |
|  | Giardia | | | |  | | | |  | | |
|  | Hook worm | | | |  | | | |  | | |
|  | Tape worm | | | |  | | | |  | | |
|  | Typhoid fever | | | |  | | | |  | | |
|  | Schistosomiasis | | | |  | | | |  | | |
|  | Guinea warm | | | |  | | | |  | | |
|  | If one or more of the above disease/s is/are selected, who is/are affected by the disease/s? | | | | | 1. Children 2. Elder persons 3. Pregnant women 4. Women 5. People with disabilities | | | | |  |
|  | What do you usually do when a member of the family is having the above disease/s? | | | | | 1. No action 2. Buy medicines 3. Go to clinic/health facilities 4. Give herbs 5. Go to traditional healer 6. Other, specify ______________________ | | | | |  |
|  | Has anyone in your household passed away (died/loss) due to the above diseases/s? | | | | | 1. Yes 2. No | | | | |  |

**I have finished my interview, thank you for your cooperation!!!**
